# Supplementary material for: The experience of pedagogical training on postgraduate rehabilitation health professionals: A qualitative study
Source: PLoS One. 2024 Dec 5;19(12):e0314920. doi: 10.1371/journal.pone.0314920 (PMC11620388; doi:10.1371/journal.pone.0314920)
Supplement: S1 Table — (PDF) [file pone.0314920.s001.pdf]

Supporting Information File 1 – The curriculum of the Master of Science in 'Rehabilitative Sciences of the Health Professions at the University of Verona

	Name:	Credits:	Hours:
1st Year	Epidemiology and Statistics	8 ECTS	200
	Fundamentals of Rehabilitation Research	7 ECTS	175
	Healthcare Management	5 ECTS	125
	Health Economics, Planning, and Law	10 ECTS	250
	Psychology of Organisational Processes	8 ECTS	200
	Applied Computer Science	2 ECTS	50
	Scientific English	3 ECTS	75
	Professional Workshops (First Year)	1 ECTS	25
	Seminar Activities (Safety in High-Risk Healthcare Environments)	1 ECTS	25
	Internship in Healthcare and Educational Institutions (First Year)	15 ECTS	375
2nd Year	Entrepreneurship in Rehabilitation Management	4 ECTS	100
	Research Methodology Applied to Work Processes	10 ECTS	250
	Tutorial Teaching Methods	8 ECTS	200
	Clinical and Organizational Advances in Rehabilitation	10 ECTS	250
	Elective Courses (Healthcare Professions)	5 ECTS	125
	Professional Workshops (Second Year)	1 ECTS	25
	Internship in Healthcare and Educational Institutions (Second Year)	15 ECTS	375
**Final Examination**			

Legend:
ECTS: European Credits Transfer and Accumulations System
