# Supplementary material for: The experience of pedagogical training on postgraduate rehabilitation health professionals: A qualitative study
Source: PLoS One. 2024 Dec 5;19(12):e0314920. doi: 10.1371/journal.pone.0314920 (PMC11620388; doi:10.1371/journal.pone.0314920)
Supplement: S2 Table — (PDF) [file pone.0314920.s002.pdf]

Supporting Information File 3 – Codes and Themes

Table 1: Theme 1 'A Brave New Pedagogical World'

Codes Leading to Theme Creation	Quotations
**The importance of good communication in pedagogy**	"I only saw a purely technical aspect, and for me, a good teacher initially was, let's say, the guru who knew everything, the ultimate expert. Now I give more importance to a teacher who can communicate effectively". (P7, 36 y, woman, physiotherapist with teaching experience)
**The importance of taking care of students**	"The thing that I appreciated about the teacher was their ability to guide us to connect with each individual in the class". (P14, 52 y, man, physiotherapist in a coordinating role with teaching experience)<br><br>"As a student, I felt important to the teachers, as they paid particular attention to make me feel like this" (P6, 34 y, Man, physiotherapist with teaching experience)<br><br>"What I liked most [...] is the fact that they made the student feel comfortable […]. You didn't have the anxiety of taking notes, and you didn't have the anxiety of having to listen 100% because all the material provided was essential to understand the lesson completely" (P2, 48 y, physiotherapist in a coordinating role)
**The importance of a consistent and good model**	"The remarkable coherence and alignment between the explanation and demonstration of Professor X were very impressive. The consistency between the content and the delivery method was particularly impactful. It was a powerful demonstration of how effective teaching can truly make a difference" (P7, 36 y, woman, physiotherapist with teaching experience)<br><br>"Particularly in the teaching methodology lessons, I found a strong sense of involvement. I noticed a remarkable alignment between the theoretical concepts presented and the practical implementation of those concepts. It was a "wow" moment for me as a student, and I felt that the professors went above and beyond to consider us as individuals. This positive experience exceeded my expectations and left a lasting impression" (P10, 29 y, woman, speech therapist with clinical experience)<br><br>"In other words, beyond understanding the purpose of this course, there is a strong experiential aspect to it, such that to understand how a good teacher should behave, I must necessarily have the experience of such a skilled lecturer" (P6, 34 y, man, physiotherapist with teaching experience)

**The importance of playing an active role in learning**	"I would say that the thing that satisfied me the most was the opportunity to get involved during the courses and to experiment directly there". (P16, 28 y, woman, speech therapist with teaching experience)<br><br>"I enjoyed the part on all the Active Learning methodologies that were presented, such as the case method, because actually experiencing just a few of them opened up a world for me, with things that I didn't know about and are certainly fascinating" (P10, 29 y, woman, speech therapist with clinical experience)<br><br>"It required me always to be very involved, very participatory. I mean, it wasn't enough for me to just listen to a lecture, it wasn't enough for me to listen to Professor X speak, but I was always asked to participate actively, building blueprints, building lessons, so that my participation was certainly an important aspect within the lesson..." (P11, 34 y, woman, physiotherapist with tutoring experience)<br><br>“Next to the student, there is the green dot, meaning you're active, you're on the ball, you're present, that is, you can't experience it passively, but actively. You have to be proactive. You have to be involved. This experience can't turn you off” (P6, 34 y, man, physiotherapist with teaching experience)
**Awareness of the behind the scene**	"I realised how challenging and complicated it can be to build a lecture, to do a good teaching job” (P15, 27 y, woman, physiotherapist with teaching experience)<br><br>"Never would I have imagined that there was so much work involved in designing a course or a module and that there would be a correspondence between the evaluation method and the content and objectives. All of this work was completely unknown, and I was delighted" (P7, 36 y, woman, physiotherapist with teaching experience)<br><br>"It's not that simple even for the teacher because it still requires preparation and takes time beforehand and effort during the class" (P8, 50 y, woman, physiotherapist and university tutor)

**Discovering how pedagogy can be fascinating**	"I didn't have any particular expectations, but I found this module very effective, and it sparked an interest in teaching in me. I'm not currently teaching courses, but it activated something in me. In other words, an interest in something I didn't think was for me" (P17, 27 y, woman, psychiatric rehabilitation therapist with clinical experience)<br><br>"I started my master's degree without any expectations, but thinking that the teaching field was the least interesting to me. After attending these two courses, I discovered that it was what I liked the most" (P15, 27 y, woman, physiotherapist with teaching experience)<br><br>"I also began the master's degree without any specific expectations, but what impressed me positively about these courses was the realisation that even a traditional lecture can be transformed into something relevant and engaging. The content can be made interesting and infused with relevance in a way that departs from the traditional, potentially boring approach that fails to captivate students. I appreciated this aspect of the course" (P4, 29 y, man, physiotherapist with clinical experience)<br><br>"We discovered a dynamic teaching model, in my opinion, where the teacher was genuinely interested in the students and aimed to let them experience what they were learning first-hand. As I mentioned earlier, this sparked an interest in teaching in me, something I had never considered before, and I believe this approach could be very effective in the classroom" (P17, 27 y, woman, psychiatric rehabilitation therapist with clinical experience)<br><br>"I'm an example of someone who has never done any teaching, and perhaps it's perspective I wouldn't mind exploring. It broadens one's horizon to a world that maybe one has never experienced first-hand" (P11, 34 y, woman, physiotherapist with tutoring experience)

Table 2: Theme 2 'Become a Cutting-Edge Lecturer'

Codes Leading to Theme Creation	Quotations
**Knowledge of adult learning mechanisms**	"How the adult learns, how the adult discovers, there is a need to make students aware of this.. It is a key that opened a door" (P3, 28 y, woman, physiotherapist with tutoring experience)<br><br>"The attention that can be brought to the different learning modalities of students, taking into consideration that each of us has a different learning style and the teacher can meet them all" (P16, 28 y, woman, speech therapist with teaching experience)
**How to tailor lecture to students**	"I tried to modify the lessons by giving something concrete, something that was important in clinical practice, in their daily lives" (P8, 50 y, woman, physiotherapist and university tutor)<br><br>"The role of the teacher has changed […] saying that they become at the service of the students is a bit too strong because the relationship between the teacher and the students is always asymmetric, but certainly this asymmetry decreases dramatically after attending this course, discovering the concept of relatedness: the need to be close to the people who are important in our lives, so even teachers have to reduce this distance by being there" (P6, 34 y, man, physiotherapist with teaching experience)
**How to evaluate students properly**	"I kept the oral exam but modified how I prepared it, creating a blueprint following Bloom's taxonomy. And so I went from knowledge to comprehension, to clinical reasoning, something higher and more advanced, and also defining scores, declaring everything during the learning contract; this learning contract that I didn't understand at the beginning of the MSc, but now I ended up using a lot" (P8, 50 y, woman, physiotherapist and university tutor)<br><br>"The other aspect that I used was the methodology for constructing the exam and especially how to correct it fairly, which was something that worried me a bit" (P16, 28 y, woman, speech therapist with teaching experience)<br><br>"I completely changed my way of providing feedback to the students. So, from a practical point of view, having a method for evaluation was truly useful" (P3, 28 y, woman, physiotherapist with tutoring experience)
**How to use active learning tools**	"I started using group projects, which are an element that I had never used before" (P3, 28 y, woman, physiotherapist with tutoring experience)<br><br>"The active participation of the students, which can also be active participants in frontal teaching" (P8, 50 y, woman, physiotherapist and university tutor)<br><br>"I created a PBL, which I never thought of". (P8, 50 y, woman, physiotherapist and university tutor)<br><br>"I tried to use group project, individual readings, videos" (P9, 33 y, woman, physiotherapist and university tutor)

**How to communicate effectively with students**	“It was constructive because now when I prepare material for the students or speak in the classroom, I always feel like I have it [the lecturer of didactics methodology] like an owl behind me. While preparing the slides, I imagine him saying: "Be careful"; "Have you given all students with different learning styles a chance to learn?"; "Have you made the necessary modifications to accommodate everyone?", "How do you manage silence when you ask questions?" (P8, 50 y, woman, physiotherapist and university tutor)<br><br>“Now we are back to frontal lecturers. However, we can still use the online platforms [they used during the covid-19 pandemic] as they are available to create a faster connection with the students. They are useful to put material, chat with students not only via email but also in a slightly more informal way” (P8, 50 y, woman, physiotherapist and university tutor)<br><br>“It is the tone of voice, the words I use. They are fundamental not only for being a teacher but also in the therapeutic relationship [with the patient]” (P3, 28 y, woman, physiotherapist with tutoring experience)<br><br>“I tried to use a learning management system in a more communicative way rather than just as a support. Another thing that I struggled with but told myself I had to do was standing next to the students during lectures. It was tough because I never thought I would have to expose myself so much. I imagined myself sitting at my desk with the computer before me and simply presenting the slides. However, as I said to myself, you have to stand up, move around, use the pointer, use the microphone, and that was an effort, but... the class started following and participating, and I understood that how I convey messages is important” (P9, 33 y, woman, physiotherapist and university tutor)
**How to arrange pedagogical tools (e.g., slides, pantry)**	“Every time I have to do a keynote presentation, or when I think about it, I always think "Okay, don't use that thing, put images, something catchy" and then it's something that stuck with me because I thought maybe the presentations I used to do were a bit boring, and now I'm able to build them better” (P14, 52 y, man, physiotherapist, coordinator with teaching experience)<br><br>“I can apply skills that I’ve learnt, especially during the course didactic methodologies, regarding PowerPoint as […] I saw how a more careful, captivating, and attention-grabbing presentation is much more effective. As a result, I received many positive feedbacks” (P17, 27 y, woman, psychiatric rehabilitation therapist with clinical experience)<br><br>“How to create slides, what characteristics they must have to be successful” (P1, 26 y, woman, speech therapist with teaching experience)<br><br>“I didn't have the instruction booklet to lecture, namely, that package of information from attending these courses” (P6, 34 y, man, physiotherapist with teaching experience)

**How to project effective lessons and educative events**	"Some concrete examples in my case, regarding the design of a 24-hour module, in the first year of the first semester of the physiotherapy degree course, having access to the tools the teachers provided us was fundamental in managing this module"(P9, 33 y, woman, physiotherapist and university tutor)<br><br>"I found myself having to design a teaching module at university, so I am reviewing the notes from these lectures [on didactics]" (P1, 26 y, woman, speech therapist with teaching experience)<br><br>"It was useful [to attend lectures in didactics] to have the skills to speak with educational providers to organise training sessions for health professionals, as well as conferences" (P5, 31 y, man, physiotherapist with clinical experience)<br><br>"I had the opportunity to experiment with my learning as they asked me to design a bioethics course. Therefore, the opportunity to experiment with the design of this course, right after the didactics courses, was nice because I could put into practice what I was learning" (P4, 29 y, man, physiotherapist with teaching experience)<br><br>"Skills related to effective presence during a lesson, the ability to cultivate relevance in the taught subject, the preparation of engaging materials such as slides and visual aids, and constructing the lesson to avoid monotony and boredom. Understanding the importance of keeping students engaged throughout the session and implementing strategies to stimulate their interest, such as inserting interactive elements or concluding with a quiz" (P1, 26 y, woman, speech therapist with teaching experience)<br>"It allowed us to understand how important it is to start with good planning to achieve an excellent result" (P16, 28 y, woman, speech therapist with teaching experience)

Table 3: Theme 3 'Something Beyond Pedagogy'

Codes Leading to Theme Creation	Quotations
**Teamwork skills**	"The exam with the group work was the one that offered me the best opportunity for collaboration" (P16, 28 y, Woman, speech therapist with teaching experience)<br><br>"We were all moving towards the same direction" (P2, 48 y, man, physiotherapist in a coordinating role)<br><br>"The group work allowed us to work as a team" (P5, 31 y, man, physiotherapist with clinical experience)<br><br>"It was the experience of something new but in a group, with other professionals, with different experiences and backgrounds that provide something enriching and formative" (P17, 27 y, woman, psychiatric rehabilitation therapist with clinical experience)<br><br>"I had a change in perspective, in the sense that it affected my work and extended to extracurricular activities. It allowed me to interact with people from different fields with unique skills, such as administrative or training responsibilities. These topics may be unrelated to physiotherapy but still have cross-disciplinary relevance. This module focused on teaching methods, enabling us to communicate using a common language." (P5, 31 y, man, physiotherapist with clinical experience)
**How to learn from experience**	"I found new meaning in my past experiences as a student, reflecting on them years later" (P11 y, 34, woman, physiotherapist with tutoring experience)<br><br>"The didactics courses allowed us to learn how to learn" (P13, 25 y, woman, speech therapist with clinical experience)<br><br>"The more there is a knowledge gap, the more there is a possibility of learning and making progress together" (P14, 52 y, man, physiotherapist, coordinator with teaching experience)
**A new awareness enhancing an open mindset**	"It's a turning point, a real shift. Something that can change your mind, change how you interpret reality and what you are doing" (P6, 34 y, man, physiotherapist with teaching experience)<br><br>"You see things from a different point of view" (P14, 52 y, man, physiotherapist, coordinator with teaching experience)<br><br>"New horizons because it gave me new perspectives for my work, because new ideas, new things came to my mind that I would like to do thanks to what I learned in the didactics courses" (P15, 27 y, woman, physiotherapist with teaching experience)

**Increasing the desire to do something well**	"A course that gives you the desire to do well." - (P3, 28 y, woman, physiotherapist with tutoring experience)<br><br>"A desire to do [...] I came home, and then in the evening I saw my friends, I said 'ah today I did the most beautiful lesson of my life, in the sense that it gave me the desire to do well" (P3, 28 y, woman, physiotherapist with tutoring experience)<br><br>"After completing these didactic courses, I felt prepared to embark on a new learning journey, independent of my job level" (P2, 48 y, man, physiotherapist in a coordinating role)
**Enhancing willingness to change for good**	"I found the enthusiasm again that […] it is the change that I am trying to motivate in all around, starting from myself." (P14, 52 y, man, physiotherapist, coordinator with teaching experience)<br><br>"I take with me the desire to continue, to learn, to change" (P2, 48 y, man, physiotherapist in a coordinating role)<br><br>"Experiencing teamwork and pushing oneself out of the comfort zone are common themes throughout these courses. The value lies in the opportunity to try new things as a team with professionals from diverse backgrounds and experiences. These collaborations result in enriching and formative experiences" (S16, 28 y, woman, speech therapist with teaching experience)<br><br>"For me, it was just one more small step towards the change that is still happening, and that is taking place. These didactics courses were part of this process. I felt like I had found a new path, a new, very fulfilling possibility … the revolution" (P4, 29 y, woman, physiotherapist with clinical experience)
**How to become a critical thinker**	"As healthcare professionals, we are perpetual learners, constantly seeking to expand our knowledge and skills through further education. However, having completed these didactic courses, we are now equipped with a new level of awareness that will allow us to approach future courses more discerningly. We will be able to delve deeper into our studies, engage with our teachers more informedly, and make more comprehensive assessments of the material presented. These courses have been invaluable in cultivating our critical thinking abilities" (P7, 36 y, woman, physiotherapist with teaching experience)<br><br>"A critical thought on the method, on the course, on the teacher, on the approach that the course you are attending has, as well as a critical thought on how you are experiencing that course" (P11, 34 y, woman, physiotherapist with clinical experience)<br><br>"From now on, I will try to see the people who will be my teachers in a seminar with a slightly more critical eye" (P1, 26 y, woman, speech therapist with teaching experience)

**Applying communication skills in clinical, private life, and working settings**	"These courses have been particularly useful not only for teaching but also in managing relationships with patients. The concepts learned in these courses have been applied to therapeutic education, health education, and more. Through this application, professionals realise they can be more effective, precise, and prompt in communicating this knowledge to their patients, not just students" (P6, 34 y, man, physiotherapist with teaching experience)<br><br>"The things that I have learned in these courses, such as evaluation skills and how to approach other people, are useful for me as a coordinator" (P15, 27 y, woman, physiotherapist with teaching experience)<br><br>"This course has equipped me with excellent tools to explain complex concepts to non-experts. Additionally, beyond its direct application in the workplace, the methodologies learned in the course, particularly in terms of relevance, can also provide insights into interpersonal communication outside of work. Understanding that what you say should interest others can be valuable for improving communication with colleagues, partners, friends, and beyond" (P6, 34 y, man, physiotherapist with teaching experience)<br><br>“Certainly, in the communicative part with the patient, with parents, it was beneficial” (P10, 29 y, woman, speech therapist with clinical experience)<br><br>“I have taken some concepts from the didactics course and applied them to leading groups in my work context. For example, I work with people who have lost their licenses due to alcohol and substance use and who may have little motivation to be there. It's essential to provide them with the educational information they need in a way that can truly capture their attention and leave them with crucial information” (P17, 27 y, woman, psychiatric rehabilitation therapist with clinical experience)<br><br>"When I communicate with patients, I often use personal examples to make them feel more connected to me. For instance, I may share my experiences or those of people close to me to explain the benefits of a certain exercise or movement. By doing this, I can make the explanation more relevant and relatable to the patient and help them see the value of our actions. I find this approach very practical and effective, and it ties in with the broader didactics themes we discussed earlier [in the focus group]. Overall, I think it's something that I should try to do more often in my practice" (P11, 34 y, woman, physiotherapist with tutoring experience)<br><br>“I also think in family relationships, or even, of course, at work with colleagues, in the relationship with others, when giving feedback, you have learned how to do it” (P1, 26 y, woman, speech therapist with teaching experience)

**Increasing patients' adherence based on their learning mechanisms**	"I transmuted this thing [what I learned during the didactics courses] into the clinical context, so exercise and physiotherapist patient, for example" (P7, 36 y, woman, physiotherapist with teaching experience)<br><br>"I found it enlightening when the lecturer compared the importance of being a motivating physiotherapist who proposes exercises that are perceived as useful and relevant to the patient, with the understanding that the characteristics of each patient play a significant role in motor learning. This made me realise that the foundations of motor control theory, which are based on motor learning, are similar to general learning principles" (P7, 36 y, woman, physiotherapist with teaching experience)<br><br>"Yes, particularly in the clinical part of the course, I found the reflection on relevance in teaching very useful. Previously, I would ask myself questions such as: "Is this activity fun for my patient/child?" or "Are they engaged?" However, now I try to take a step further and consider whether the activity is relevant for the child about their disability, fatigue, and the effort they are making. This involves a more in-depth clinical reflection on the concept of relevance that I mentioned earlier" (P10, 29 y, woman, speech therapist with clinical experience)<br><br>"The course emphasised the importance of effective communication and the need to adapt our communication style to the individual learning styles of our patients. It reminded me that everyone learns differently and that we must find ways to connect and communicate effectively with each patient" (P8, 50 y, woman, physiotherapist and University Tutor)<br><br>"I noticed how the concepts of relevance and adapting to different learning styles are applicable not only in a didactic context but also in my clinical practice. Working with patients with developmental age and learning disabilities, I see that the teaching style used with them often clashes with their learning style, leading to difficulties in learning. This has activated my thoughts on the importance of adapting to different learning styles, a concept I find applicable to my everyday work with patients" (P16, 28 y, woman, speech therapist with teaching experience)
