# Supplementary material for: The experience of pedagogical training on postgraduate rehabilitation health professionals: A qualitative study
Source: PLoS One. 2024 Dec 5;19(12):e0314920. doi: 10.1371/journal.pone.0314920 (PMC11620388; doi:10.1371/journal.pone.0314920)
Supplement: Attachment — Submitted filename: PO_Rebuttal Letter.docx [file pone.0314920.s004.docx]

**Authors’ general comment:**

We would like to thank the Editor and the reviewers for their comments that improve the quality of our work. Please, find our rebuttal letter where we have highlighted in yellow all the amendments to our paper.

**Reviewer #1**

**Reviewer’s general comment:**

Comments to the Author:

This manuscript has very important evidence that provide evidence on the impact of such a kind of trainings on the quality of practices in service provision and education. To make sure your knowledge reach to the global and local community who can benefit form this evidence, I strongly recommend you consider the revision recommended to improve your result and discussion presentations. Finally, it will be great to have a language support to improve the presentation. Thank you!

**Authors’ reply:** We thank the reviewer for their positive feedback on the importance of our study’s evidence. We have carefully addressed each of the reviewer’s suggestions.

Abstract

Comments to the Author:

Line #35+ Result: Participants description and the finding could be improved with better presentation as the current one is confusing. E.g. …with average age 33 ± 9 and majority women (n=12). Three themes were developed: 1) “A Brave New Pedagogical World” …. 2) ….. and 3) ….

**Authors’ reply:** We thank the reviewer for their valuable feedback. We have revised the Results section of the abstract to improve the clarity of the participant description and findings presentation. The participants’ demographics are now stated more clearly, and the key themes are presented in a simplified format to enhance readability

**Authors’ action:**

**Page 2, lines 35-39:** Three focus groups were conducted with seventeen Italian participants (age: 33 ± 9; 71% women, n=12; 29% men, n=5). The analysis identified three main themes: 1) “A Brave New Pedagogical World,” reflecting participants' exposure to innovative teaching approaches; 2) “Becoming a Cutting-Edge Lecturer,” highlighting skills acquired for delivering inspiring lectures; and 3) “Something Beyond Pedagogy,” where participants reported skills applicable to other professional contexts, including clinical practice.

Line #40+ Discussion: I recommend replacing ‘discussion’ by ‘conclusion’ as we are not actually discussing at the abstract. Also, this part states a kind of recommendation to implement this education. However, the study doesn’t test about the impact of this training on the impact of the training their education practice. Hence, we don’t have evidence to say it is effective and recommend for implementation at large scale. I think, there is another stage next to this study, evaluation of the impact of this training.

**Authors’ reply:** We thank the reviewer for their insightful comments. We have revised the abstract to replace ‘Discussion’ with ‘Conclusion,’ as suggested. Additionally, we have adjusted the language in the conclusion to avoid suggesting large-scale implementation, emphasizing instead the potential implications of this training for further research.

**Authors’ action:**

**Page 2, lines 40-43: Conclusion**: The results of this study showed that pedagogical courses provide a positive learning experience for rehabilitation health professionals, helping them develop relevant pedagogical skills. Although our findings suggest the potential benefits of these courses in preparing healthcare professionals for teaching roles, further studies are needed to evaluate their direct impact on educational practices and patient outcomes.

Introduction
Line #48-53: You set a foundation, but would make more sense to add a context where the existing education system is impacting the quality of graduates (in their service – be it in clinical or education practices) so as to justify the need to improve the pedagogical skills of educators could add value to the quality of graduates.

**Authors’ reply:** We thank the reviewer for this helpful suggestion. To provide a clearer justification for the need to enhance pedagogical skills among healthcare educators, we have expanded the Introduction to include context on how the current education system affects graduate quality, particularly in clinical and educational practices.

**Authors’ action:**

**Page 3, lines 51-56:** Health professionals who act as lecturers in higher education necessitate the acquisition of pedagogical skills to enhance the efficacy of their lectures [1]. Being a clinical expert does not necessarily mean being an expert educator. Most educators in the medical field did not receive adequate training in pedagogy [1]. Without specific training, they resorted to appealing to their personal experience, using those patterns they experienced as students through a 'modelling' process and relying on their cultural sensitivity and capacity for elaboration [2]. As healthcare services evolve and become more complex, it is essential for educators to go beyond such ‘modelling’ processes [3]. Graduates in health professions are now expected to possess clinical expertise and engage in evidence-based practices, following patient-centred care [4]. Educators may struggle adequately preparing students for these multidimensional demands without structured pedagogical training. Therefore, configuring a well-defined pedagogical training programme for new lecturers is crucial to support high-quality graduate outcomes in both clinical and educational practices [1].

Line #60+: You could provide the Italian context of what is happening and what is know about the current practice.

**Authors’ reply:** We thank the reviewer for their suggestion. We have expanded this section to include the current context in Italy, describing the state of pedagogical training and any specific requirements or initiatives relevant to health professionals in higher education

**Authors’ action:**

**Page 3, lines 65-72:** In Italy, structured pedagogical training for healthcare professionals is less standardised than other European countries [8]. While some universities offer pedagogical courses within specific health programmes, a national framework or requirement for pedagogical certification is not yet established or needed to teach in higher education. This creates variability in teaching quality across institutions [9]. The Master of Science (MSc) in 'Rehabilitative Sciences of the Health Professions' is an example of a postgraduate programme aiming to bridge this gap. Established with a unique curriculum that includes courses in pedagogical methodology, curriculum design, and tutorial methodology, this MSc programme attracts a diverse range of students who obtained a Bachelor of Science (BSc) in one of the eight rehabilitation health professions recognised by the Italian health professional register [10–12].

Line #70: The statement “meanings attached to education are influenced by ethnicity, living area, and working background” require citation.

**Authors’ reply:** Thank you for your constructive feedback regarding the statement that “meanings attached to education are influenced by ethnicity, living area, and working background.” I appreciate your attention to this matter. In response, I have added relevant citations to the manuscript that underscore how systemic factors such as race, ethnicity, socioeconomic status, and geographic location shape educational experiences and perceptions.

Line #78-79: The statement “The study complied with the Declaration of Helsinki” need be linked with the its purpose/function like the way you described after the connector “and”

**Authors’ reply:** Thank you for your insightful comment regarding the connection between the study's compliance with the Declaration of Helsinki and its ethical implications. I have revised the text to clarify that adherence to the Declaration was intended to ensure the protection of participants' rights and welfare throughout the research process. This emphasizes the importance of ethical standards in qualitative research. Thank you for your feedback, which has helped improve the clarity of this section.

**Authors’ action:**

**Page 4, lines 88-90:** The study complied with the Declaration of Helsinki, ensuring ethical standards were upheld to protect the rights and welfare of participants, and was reported according to the Consolidated Criteria for Reporting Qualitative Research (COREQ).

Line #84+, Participants description: Some of the partis in the first paragraph could be moved to provide a context about Italy in the introduction section, but with citation.

**Authors’ reply:** We appreciate your insightful feedback regarding the participant description. In response, we have restructured the introduction to include relevant information about the context of Italy's pedagogical training for health professionals. This adjustment provides a clearer framework for understanding the significance of our study and the diverse backgrounds of the participants in the Master of Science program. We have ensured that citations are included to support the contextual information presented. This modification not only aligns with your suggestion but also enhances the overall coherence of the paper. Thank you for your valuable input.

**Authors’ action:**

**Page 3, lines 65-72:** In Italy, structured pedagogical training for healthcare professionals is less standardised than in other European countries [8]. While some universities offer pedagogical courses within specific health programmes, a national framework or requirement for pedagogical certification is not yet established or needed to teach in higher education. This creates variability in teaching quality across institutions [9]. The Master of Science (MSc) in 'Rehabilitative Sciences of the Health Professions' is an example of a postgraduate programme aiming to bridge this gap. Established with a unique curriculum that includes courses in pedagogical methodology, curriculum design, and tutorial methodology, this MSc programme attracts a diverse range of students who obtained a Bachelor of Science (BSc) in one of the eight rehabilitation health professions recognised by the Italian health professional register [10–12].

**Page 4, lines 97-102:**  Recent graduates and students of the MSc. in 'Rehabilitative Sciences of the Health Professions at the University of XXX (XXX, XXX) were recruited. This MSc focused on research methods and methodologies, teaching in higher education and health economics. This MSc programme focuses on research methods, pedagogy in higher education, and health economics. It includes a unique curriculum with mandatory courses such as pedagogical methodologies, curriculum design, and tutorial methodology. The programme attracts a diverse cohort of students with a BSc in one of the eight rehabilitation health professions recognised by the Italian health professional register, including physiotherapists, speech therapists, occupational therapists, orthoptists, podiatrists, psychiatric rehabilitation technicians, psychomotor therapists, and professional educators. All courses within the degree program's curriculum are mandatory, and the complete course curriculum can be viewed in the Supporting Information file 1.

Line #86-87: The sentences that started with “This MSc…” doesn’t flow well. Your want to indicate the structure and contents of the education program, but it needs improvement.

**Authors’ reply:** Thank you for your feedback regarding the clarity of the sentences about the MSc programme. We have already revised this sentence while addressing your previous comment to enhance its readability and overall flow.

Line #96: The statement “purposely sampled to identify those who were more informative to answer the research question”, what criteria in your sampling process were used to identify those who are “more informative to answer the research question”? Some details about the criteria would add values.

**Authors’ reply:** Thank you for your valuable feedback regarding the clarity of our sampling process. We appreciate your request for additional details about the criteria used to identify participants who were considered "more informative" for addressing the research question. In our revised manuscript, we have elaborated on the specific criteria employed during participant selection. We focused on individuals' relevant experiences in rehabilitation and education, which included their professional backgrounds (such as physiotherapy and speech therapy), as well as their levels of lecturing experience. Additionally, we considered participants’ roles in coordinating staff and departmental operations, as well as their experiences as clinical tutors, to ensure a comprehensive understanding of pedagogical courses.

These criteria were designed to capture a diverse range of perspectives, enhancing the richness of our qualitative data and ensuring that we could explore the educational experiences of health professionals from various rehabilitation disciplines.

**Authors’ action:**

**Page 4, lines 106-120:** Participants were purposely sampled to identify those who were more informative to answer the research question by ensuring that the group was as heterogeneous as possible [20]. To achieve this, we sought participants with relevant experience in rehabilitation, education, and management. Specifically, the authors contacted the course leader of this MSc, asking her to reach out to the former or current students of the programme based on different criteria. The course leader contacted the students via email. She sent an email outlining the aim of the study, its design and the informed consent to be signed. She specifically explained in the email to contact BM instead of her to programme the focus groups. This decision was made to prevent the course leader from knowing which students participated in the focus groups, as she taught a course related to pedagogical methodologies. Participants were recruited based on their professional backgrounds, such as physiotherapy, speech therapy, and others, to ensure a balanced representation of various rehabilitation areas. Additionally, we included professionals with varied levels of lecturing experience (or none), those with roles in coordinating staff and departmental operations, and clinical tutors who work closely with students. Individuals with expertise coordinating staff and departmental operations alongside clinical tutors were specifically included to capture insights on the course's impact from professionals accustomed to managing team dynamics and mentoring students in clinical settings. Their perspective was deemed valuable for assessing the development of pedagogical and interpersonal skills relevant to educational and clinical practice. This approach aimed to capture a broad lens of experiences related to the pedagogical courses.

Line #97-98: Same comment as above that is about “This MSc…”

**Authors’ reply:** We have already revised this sentence while addressing your previous comment.

Line #101-103: The point describe in this sentence is not convincing as relevant to the research questions as these roles are not specific to educational experience.

**Authors’ reply:** Thank you for your comment regarding the inclusion of coordinators and clinical tutors to assess the course’s impact. We understand your observation that these roles may not be directly tied to formal educational experience; however, we believe their perspectives add valuable depth to our study. Their responsibilities in coordinating staff, managing departmental operations, and mentoring students involve frequent interaction with students and academic personnel, providing them with unique insights into how pedagogical training affects teaching and learning dynamics. As our analysis shows—particularly under the theme “Something Beyond Pedagogy”—these roles foster skills that are transferable to both educational and clinical settings, enhancing our understanding of the training’s broader impact.

To clarify this rationale further, we have revised the text to better highlight why we chose to include these professionals in our sample.

**Authors’ action:**

**Page 4, lines 116-120:** Individuals with expertise coordinating staff and departmental operations alongside clinical tutors were specifically included to capture insights on the course's impact from professionals accustomed to managing team dynamics and mentoring students in clinical settings. Their perspective was deemed valuable for assessing the development of pedagogical and interpersonal skills relevant to educational and clinical practice. This approach aimed to capture a broad lens of experiences related to the pedagogical courses.

Line #125: use either the full term “focus group discussions” or FGD once you fully describe when you first use it.

**Authors’ reply:** Amended as suggested

Line #128: “At the beginning of each meeting …” seems that you have multiple meetings within a specific group. So, it is better to describe as “prior to each focus group discussions, ….”

**Authors’ reply:** Amended as suggested

Line #129: The statement “A brief presentation of each participant followed this introduction by the researchers” is confusing, so consider revision to add clarity or remove.

**Authors’ reply:** Thank you for your comment. We recognize that the sentence may have been unclear. Our intention was to indicate that each participant provided a brief introduction after the researchers summarized the course objectives. We have revised the sentence to improve clarity.

**Authors’ action:**

**Page 5, lines 148-151:** Prior to each FGD, the contents and topics covered within pedagogical courses in the MSc study's objective were summarised. After the researchers introduced the study’s objectives and summarised the topics covered in the pedagogical courses, each participant briefly introduced themselves to the group.

Line #122-139: Generally the interviewing process is too detailed, particularly since you have already included the interview guides, descriptions related to the flow of questions were a redundant point.

**Authors’ reply:** Thank you for your feedback. However, we appreciate that in qualitative research, it is crucial to provide comprehensive information about the research setting and procedural specifics to ensure transparency, reproducibility, and methodological rigor. These details allow readers to fully understand the context and structure of our focus groups, enhancing the credibility and trustworthiness of our findings. As an author group, we believe that this level of detail is necessary and aligns with best practices in qualitative research. Thank you for your understanding.

Line #143, Table 1: Consider improving the table format as the current structure is not in its best way.
Overall question on data acquisition: What was the language used to facilitate the focus group discussion (data generation process)? If you use any language other than English, what was it? When and how did you translate it to English language, providing that the manuscript is prepared in English? Who did the translation and why? I am forwarding these points as they are critical for the research rigor and for the quality of the evidence generated.

**Authors’ reply:** Thank you for your insightful comment. We recognize the importance of clarifying the language used in the focus group discussions to ensure transparency and research rigor. The focus groups were conducted in Italian, the participants’ native language, to capture their experiences as authentically as possible and to allow for the generation of themes based on their own words without the risk of misinterpretation through translation. Themes and selected quotations were then translated into English by the research team for inclusion in this manuscript. This process, conducted by bilingual researchers fluent in both Italian and English, was designed to maintain fidelity to the original meanings of participants’ responses. We have now added a paragraph to the manuscript to address this aspect explicitly. Thank you for helping us to improve the clarity and rigor of our study.

**Authors’ action:**

**Pages 5,6 lines 162-167:** The FGDs were conducted in Italian, the native language of all participants, to ensure that their experiences and perspectives were captured authentically. This approach allowed the researchers to generate themes directly from participants' words in their mother tongue, thus preserving the integrity of their responses during the analysis phase. After themes were established, selected quotes and themes were translated into English for this manuscript. The translation was performed by the research team members fluent in Italian and English to ensure that the translated content remained as faithful as possible to the participants’ original meanings.

Line #146-150: I recommend to move most of these parts to the declaration of co-authors’ contribution.

**Authors’ reply**. We believe that the characteristics of the researchers involved in data analysis are essential for understanding how data were interpreted and contextualised, as these aspects contribute to capturing the nuances of qualitative data generation. This is particularly important in RTA, where knowledge is rooted in researchers’ background. In Qualitative research, transparency about the researchers' backgrounds is as fundamental as detailing the characteristics of participants or the data collection methods. Therefore, we consider these details to be an integral part of the manuscript and they should be in the manuscript, as also reported in the COREQ.

Line #152-157, Data analysis: The reference guiding your analysis can be cited once at the beginning and then no need to repeatedly mention after each sentence in the same paragraph.

**Authors’ reply:** Amended as suggested

Line #156-157: The last statement here needs improvement for clarity, as it confuses due to citation of the table which you could have cited it at the end of the statement. Consider revision

**Authors’ reply:** Thank you for your comment. We agree that the sentence could be clarified by adjusting the placement of the citation for Table 2. We have revised the statement to improve readability.

**Authors’ action:**

**Page 7, lines 180-190:** Qualitative data were analysed using Braun and Clark's ‘Reflexive Thematic Analysis’ (RTA) to identify the patterns of meaning and consequently generate themes relating to the experiences of the pedagogical courses [23]. RTA was selected for analysing the qualitative data due to its flexibility and adaptability, which allowed us to capture the nuanced perspectives of participants in pedagogical training courses. RTA is particularly suitable for studies with an experiential focus, as it enables a deep exploration of participants' personal insights and the meanings they attach to these experiences. This approach allowed us to generate themes inductively, ensuring that they authentically reflected participants' voices without imposing pre-existing frameworks. Moreover, RTA facilitated a collaborative analysis process, enriching the rigor of theme development and aligning with the social constructionist framework of this study. From the point of view of the epistemological framework, this study adopted a social constructionist approach and an experiential orientation. The coding was mainly at a descriptive/semantic level. To analyse the focus group data, we followed the six steps of RTA as outlined by Braun and Clarke, as shown in Table 2.

Line #159, Table 2: I don’t think this content need a table as it is possible to describe the steps and actions taken at that stage, who did it, if needed.

**Authors’ reply:** Thank you for your valuable feedback on the format used to outline the six steps of Reflexive Thematic Analysis (RTA). We understand your suggestion to describe these steps narratively rather than in a table format. However, we believe that retaining the table provides significant advantages for our readers and it is a common procedure adopted in RTA. The table offers a clear and structured summary of the RTA process and the involvement of each author at each stage. This layout allows readers to quickly grasp the sequence of actions, the distinct roles of each author, and the specific tasks performed in each phase of the analysis. Given the detailed nature of our methodological approach, a table format enhances readability and enables rapid consultation, which may be particularly beneficial for readers who are familiar with RTA but are interested in the specific ways we applied it in our study.

Moreover, the table emphasises the collaborative aspects of our analysis, showing at a glance how different team members contributed to each stage. We believe that this level of transparency regarding authors' involvement aligns well with our commitment to rigour in qualitative research.

For these reasons, we would prefer to keep the table, as it maintains clarity and facilitates the reader's understanding of both the RTA process and our specific analytical approach. Thank you again for your constructive suggestions.

Results
Line #164: use either the full term “focus group discussions” or FGD once you fully describe when you first use it.

**Authors’ reply:** Amended as suggested

Line #164-165: The statement “Three focus groups were conducted in September 2023 with seventeen Italian participants” is repetition and result is not its relevant place as you have already escribed it in the methods section.

**Authors’ reply:** Thank you for your feedback. We agree that the statement regarding the number and timing of the focus groups is redundant, as this information is already provided in the *Methods* section.

**Authors’ action:**

**Page 8, lines 197-198:**  Seventeen Italian participants (average age 33 ± 9; 71% women, n=12; 29% men, n=5) took part in the FGD. Each FGD lasted about 90 minutes.

Line #164-165: See my comment above, at the abstract section, the result part for a better way of presenting the data.

**Authors’ reply:** Amended as suggested

Line #165: Delete “Table 3 reported participants’ demographic information.” As t doesn’t add value, rather you may cite the table at some point in this paragraph.

**Authors’ reply:** Amended as suggested

Across the manuscript, search the words “focus groups” and try to either write them fully as “focus group discussions” or replace with its application (FGDs)

**Authors’ reply:** Amended as suggested

Line #169: The statement “the reader will find the description of each theme with …” need revision using active voice or the whole sentence need revision “In the following subsections, the reader will find the description of each theme with a table containing the primary quotes that brought the research to generate the specific themes.”

**Authors’ reply:** Thank you for your helpful suggestion. We agree that revising the sentence to use active voice will improve clarity and readability. We have rephrased the sentence as you suggested.

**Authors’ action:**

**Page 8, lines 201-202:** The following sections describe each theme in detail, with tables in Supporting Information File 2 that highlight the primary quotes leading to the generation of each specific theme.

Across the manuscript, I would recommend to use Male/Female rather than Man/Woman.

**Authors’ reply:** Thank you for your thoughtful suggestion. We appreciate the importance of consistency in terminology across the manuscript. However, we have intentionally used "man/woman" rather than "male/female" to refer specifically to participants' self-identified gender, aligning with the current understanding that "man/woman" reflects gender identity, whereas "male/female" typically refers to sex assigned at birth. Using "man/woman" allows us to more accurately represent participants' gender identities as they expressed them in the study.

We hope this clarification addresses your concern and aligns with our commitment to respectful and precise language.

See also: <https://orwh.od.nih.gov/sex-gender>

*A person’s gender identity (e.g., woman, man, trans man, gender-diverse, nonbinary) is self-identified, may change throughout their life, and may or may not correspond to a society’s cultural expectations based on their biological sex traits. For example, a person with typical female (sex term) sex traits may or may not be a woman (gender identity). Although gender is often portrayed and understood in Western cultures using binary categories (man or woman) and is often assumed at birth based on a person’s sex traits, many cultures throughout history have recognized a diversity of forms of gender identity and gender expression (how a person communicates their gender to others through behavior and appearance).*[*^[2]^*](https://orwh.od.nih.gov/sex-gender#ref-2-foot)

Line #172, Table 3: Make sure the information about the working area couldn’t cause any ethical breaches as it can be used to relate with a specific participant by linking with the other characteristics across the document. Also, you may need to consider if this information is really relevant/would add value to the research anyways.

**Authors’ reply:** Thank you for highlighting this important ethical consideration. We agree that including information about participants' geographic working areas could potentially compromise anonymity when combined with other demographic characteristics. After careful review, we have decided to remove the geographic area information from Table 3 to ensure participant confidentiality and avoid any risk of identification. We believe this adjustment does not affect the value or relevance of the findings.

Line #173: Thee Legend “ID” may not directly identification number as it is not only a number and has a letter within the code. May be this can be “participants unique code”

**Authors’ reply:** Amended as suggested

Line #175-243: Presentation of the three themes. I can see there are three themes generated from the data and that you have presented them in detail within a table, with a number of quotes. That is a great depth, but that again makes the result to appear at very breadth level, and lack some level of depth analysis to present it as a manuscript. The paragraphs under each themes have great description and attempted to cover most of the points. However, non of them were structured as a conceptual description of the themes and sub-themes, if what you have presented in the table are sub-themes. It is highly recommended to describe them explicitly. Present each theme with their sub-themes and associated quotes need to be integrated with the presentation of your interpretation.

The tables have so much detailed quotes that can be used as additional file, if needed to provide the details, but the results need major revision to rewrite the themes and sub-themes with associated quote, probably one or two quotes could be presented, using various formats of presenting the quotes. I would strongly recommend the authors to refer other articles to learn how results are presented using quotes to support their argument/interpretation.

**Authors’ reply:** Thank you for your constructive feedback, which has guided us in refining the results section. Based on your recommendations, we have fully revised the presentation of the themes, providing a more structured and conceptual description. Specifically, we have made explicit the *codes* (now italicized in the text) that led to the construction of each theme, reflecting the underlying analytical process and ensuring clarity in how each theme was generated. These codes correspond to what you referred to as “sub-themes” in the tables.

Additionally, we have integrated selected participant quotes directly into the narrative for each theme. These quotations now serve to illustrate and substantiate the themes within the text, linking participant voices directly to the authors' interpretation and enhancing the depth of analysis. In choosing and formatting these quotes, we have aimed for a cohesive flow that aligns with recommended practices for presenting qualitative results.

Regarding the tables with detailed quotations, we appreciate the suggestion to consider them as supplementary material. In response, we have moved these tables to a new Supporting Information File 2. This allows the manuscript's main text to maintain a streamlined presentation of the results while preserving the depth and richness of the participants' perspectives for readers who wish to explore the data supporting each theme in greater detail.

Thank you again for your guidance, which has been invaluable in strengthening the presentation and clarity of our findings.

**Authors’ action:**

**Pages 8,9,10,11 lines 206-318: Theme 1: A Brave New Pedagogical World**

This theme revolves around the transformative experience participants described as encountering a “brave new pedagogical world,” shifting their understanding of teaching from a technical skill to a relational and dynamic practice. The researchers identified several codes that helped construct this theme (Supporting information File 2 – Table 1). A key aspect of this theme is *the importance of good communication in pedagogy*, where participants began to recognise that effective teaching requires expertise and the ability to communicate clearly and engage students. One participant noted, “A good teacher isn’t just a guru with knowledge, but someone who can communicate effectively” (P7, 36, physiotherapist with teaching experience). This reflection shows a shift from viewing teachers as merely content experts to seeing them as facilitators who actively shape the learning environment. Another essential element is *the importance of taking care of students*, illustrated by how participants felt valued and supported by their instructors. One participant shared that this caring approach was meaningful: "I felt important to the teachers; they made me feel comfortable and provided all the essential materials” (P6, 34, physiotherapist with teaching experience). Experiencing this level of care firsthand helped participants understand the impact of a student-centred approach, reinforcing the importance of empathy and attentiveness in pedagogy. Participants also highlighted *the importance of a consistent and good model* in teaching, where the alignment between teachers’ actions and their pedagogical principles served as an inspiring example. One participant observed, “The remarkable coherence between what Professor X explained and how they taught was impressive,” adding that this consistency created a “wow” moment that left a lasting impression (P10, 29, speech therapist with clinical experience). This experience emphasised how leading by example in teaching methodology could profoundly influence students’ perceptions of teaching. The theme further includes *the importance of playing an active role in learning*. Rather than absorbing information passively, participants were encouraged to be active creators, directly engaging with the material through hands-on tasks and collaborative projects. As one participant described, “The active learning methodologies we used opened up a whole new world for me” (P10, 29, speech therapist with clinical experience). This code reflects a shift toward understanding learning as a participatory process, deepening participants’ appreciation for methods that invite students to actively engage and take ownership of their learning. Participants also expressed *an awareness of the behind-the-scenes effort in teaching* as they came to realize the extensive planning, organisation, and commitment required to create effective lessons. One participant reflected, “I realised how challenging and complicated it can be to build a lecture” (P15, 27, physiotherapist with teaching experience). This insight fostered a newfound respect for teaching as a complex and labour-intensive discipline, going beyond what they initially expected. Finally, the theme encompasses *discovering how pedagogy can be fascinating*, as some participants reported developing a genuine interest in teaching. For instance, one participant noted, “I never imagined that teaching could be so fascinating. I started with no expectations, but by the end, I felt an unexpected interest in becoming a lecturer” (P15, 27, physiotherapist with teaching experience). Collectively, these reflections illustrate how the experience opened up new perspectives for participants, expanding their view of teaching as not just a skill but a meaningful and inspiring profession.

**Theme 2: Becoming a Cutting-Edge Lecturer**

This theme reflects participants’ desire to become “cutting-edge lecturers” who integrate technical expertise with dynamic, student-centred teaching strategies to enhance learning outcomes. The researchers identified several codes that contributed to constructing this theme (Supporting information File 2 – Table 2). A foundational code within this theme is *knowledge of adult learning mechanisms*, which many participants found transformative. Participants expressed how understanding the distinct learning needs of adults allowed them to adapt their teaching styles more effectively. One participant noted, “The course showed us how adults learn and the importance of meeting different learning styles” (P3, 28, physiotherapist with tutoring experience), emphasising that this insight opened new possibilities for crafting lessons that resonate with adult learners.

Another significant code, *how to tailor lectures to students*, illustrates participants’ growing commitment to making lessons relevant to students' real-world experiences. Participants highlighted how they adjusted their lectures to integrate practical examples, thereby connecting theoretical content with clinical applications. As one participant shared, “I tried to modify my lessons, bringing in concrete examples from clinical practice” (P8, 50, physiotherapist and university tutor), which helped them reframe their role as facilitators who guide students in applying knowledge to practical settings. *How to evaluate students properly* emerged as another key component, where participants embraced assessment frameworks such as Bloom’s taxonomy to foster critical thinking and clinical reasoning. One participant described the changes in their assessment approach, stating, “I redesigned my exams to encourage clinical reasoning, and I use a learning contract to set clear expectations” (P8, 50, physiotherapist and university tutor). This approach aimed to promote transparency and fairness, contributing to a more equitable and supportive learning environment. Participants also emphasised *how to use active learning tools* to create interactive and engaging experiences. They described experimenting with methods like group projects, videos, and individual tasks to make lectures more participatory and impactful. “I used group projects, videos, and individual tasks to make lectures interactive,” one participant explained, reflecting on the benefits of these strategies in making learning more dynamic and student-centred (P9, 33, physiotherapist and university tutor). *How to communicate effectively with students* was identified as an essential skill in capturing and maintaining student attention. Participants discussed how adopting effective communication techniques, such as modulating tone, using movement, and incorporating digital tools, enhanced classroom engagement. One participant emphasized, “It’s not just what you teach but how you convey it. Using tone, movement, and digital tools helped me keep students engaged” (P9, 33, physiotherapist and university tutor), underscoring the importance of adaptability and presence in fostering a vibrant learning environment. Finally, *how to arrange pedagogical tools and project effective lessons* captures participants’ increased proficiency in structuring and planning educational content. They learned to design lessons thoughtfully, ensuring that each component was aligned with learning objectives and tailored to students’ needs. One participant remarked on this approach: “Every time I have to present, I think about making the presentation captivating, with images and elements to keep attention high” (P14, 52, physiotherapist and coordinator with teaching experience). This planning allowed participants to envision themselves as forward-thinking educators, ready to inspire and connect with students in meaningful ways.

Together, these codes capture the essence of becoming a “cutting-edge lecturer” by focusing on preparation, adaptability, and a strong commitment to student-centered teaching.

**Theme 3: Something Beyond Pedagogy**

This theme explores how the pedagogical skills participants gained extended beyond the classroom, significantly enriching their clinical practice and personal lives. The researchers identified several codes that contributed to constructing this theme (Supporting information File 2 – Table 3). The first code, *teamwork skills*, captures how participants developed a stronger appreciation for interdisciplinary teamwork through collaborative projects. This experience enhanced their ability to communicate and engage constructively with colleagues from diverse backgrounds. As one participant reflected, “The group work allowed us to learn from each other and work together toward a common goal” (P16, 28, speech therapist with teaching experience). This collaborative experience strengthened their professional relationships and built a foundation for effective teamwork in various settings. Another core code is *how to learn from experience*, highlighting the participants’ newfound appreciation for self-assessment and growth through reflection. This reflective mindset helped participants approach new challenges with openness and adaptability, as one noted: “The courses encouraged me to reflect on my actions and ask questions, which helps me approach new challenges with an open mind” (P11, 34, physiotherapist with tutoring experience). This emphasis on reflection was seen as critical for both professional development and personal growth. *Developing a new awareness that enhances an open mindset* emerged as an important outcome, as participants expressed a renewed willingness to embrace change and challenge themselves. One participant described this transformation, stating, “The course has inspired a desire to continue, to learn, and to change” (P2, 48, physiotherapist in a coordinating role). This mindset shift was accompanied by *an increased desire to do things well*, with participants describing how the course sparked a motivation to pursue excellence in their work. One participant explained, “I came out of the class wanting to do better; it gave me the desire to strive for quality” (P3, 28, physiotherapist with tutoring experience). This drive was seen as foundational to their ongoing personal and professional development. The code *enhancing willingness to change for good* further underscores how the training inspired participants to embrace continuous improvement. This growth-oriented perspective encouraged them to view challenges as opportunities for personal and professional advancement, fostering resilience and adaptability in their careers. *Becoming a critical thinker* was another key code, where participants learned to approach training and educational methods with a discerning eye. This critical perspective empowered them to evaluate teaching methods more effectively and prioritize those most beneficial to learners. “These courses have taught me to look critically at teaching methods and select what truly benefits students,” explained one participant, underscoring how this skill would support their future development (P1, 26, speech therapist with teaching experience). *Applying communication skills in clinical, personal, and professional settings* was also transformative for participants. They reported that improved communication made their interactions with patients, colleagues, and family members clearer and more impactful. One participant shared, “I’ve applied what I learned in didactics to better communicate with patients and colleagues, making my interactions clearer and more impactful” (P6, 34, physiotherapist with teaching experience). This adaptability helped participants build rapport and understanding across diverse interactions. Lastly, *increasing patient adherence based on their learning mechanisms* reflects how participants transferred their pedagogical insights into clinical practice. Recognising that tailoring communication to patients’ individual learning styles could boost engagement, one participant remarked, “Knowing how to adapt my approach based on learning styles has improved my interactions with patients, especially in motivating them to follow through on exercises” (P7, 36, physiotherapist with teaching experience). This application of pedagogical principles to patient care underscores the relevance of these teaching skills in clinical settings, ultimately enriching participants’ ability to connect with and motivate their patients. Collectively, these codes reveal how the skills gained in pedagogical training extended beyond the classroom, enabling participants to enhance their effectiveness in clinical practice, strengthen their personal and professional relationships, and adopt a mindset of continuous learning and adaptability.

Discussion
Line $245+: The discussion section is well written, but authors attempted to discuss each piece of the information generated in the result section. Rather, I would recommend stepping back and see which points are most outstanding and deserve a focused discussion/argument to elevate the interpretation. This again will be linked to the earlier comment on the result section that require major revisions on its content. Therefore, I would recommend first to step back to the result section, complete the necessary revisions and come back to the discussion with the most powerful findings that you want to discuss with depth and made a conclusion on your study.

**Authors’ reply:** Thank you for your thoughtful feedback on our discussion section. We appreciate your recommendation to prioritise the most prominent findings for a more focused discussion. After revisiting our analysis, we understand your suggestion and the value of highlighting core insights; however, we believe that each finding identified in our results contributes uniquely to the broader understanding of our study topic.

In developing the discussion, our aim was to ensure a comprehensive interpretation by addressing all aspects of our results, given that each theme and code offers distinct insights and practical implications. We found that discussing each result allowed us to explore the depth and range of participants' experiences fully. We carefully structured the discussion to avoid redundancy and maintain clarity, ensuring that each point builds toward an integrated understanding of the pedagogical impact.

With this approach, we aim to provide readers with a holistic view, capturing the nuances that emerged from our data. We hope this rationale aligns with our study’s objectives and enhances the value of our contributions to the field. Thank you again for your invaluable guidance, which has helped us refine both the results and discussion sections.

Acknowledgements
Line #367-368: I think you have participants who had invested their time and hence deserved to be acknowledged.

**Authors’ reply:** Thank you very much for your thoughtful feedback regarding the acknowledgment of the participants in our study. We appreciate your attention to this important aspect, and I fully agree that those who contributed their time and efforts deserve to be recognized.

**Authors’ action:**

**Page 15, lines 451-454: Acknowledgments**

We wish to extend our sincere gratitude to all participants of this study. Your invaluable contributions and willingness to dedicate your time to this research have significantly enriched our findings. This work would not have been possible without your involvement.

**Reviewer #2**

**Reviewer’s general comment:**

The Article is well written and all the aspects are well explained. There sufficient information gathered to support the need and the significance of the the study. Methodology is well written to the extent of replication. The results of all the objectives have been met and illustrated. Discussion sound adequate and cover all the aspects.

**Authors’ reply:**

Thank you very much for your positive feedback and encouraging comments. We are pleased that you found the article well-written, with clear explanations across all aspects of the study. We are also glad to hear that the methodology provides sufficient detail for replication and that the results adequately meet the study’s objectives. Your acknowledgment of the thoroughness of our discussion is greatly appreciated. Thank you again for your constructive review and support.

**Reviewer #3**

**Reviewer’s general comment:**

Consider reorganizing the introduction to follow a more logical flow: starting with the general need for pedagogical skills among health professionals, followed by the existing strategies in different European countries, and then narrowing down to the specific gap concerning rehabilitation professionals in Italy.

**Authors’ reply:** Thank you for your valuable suggestion regarding the organization of the introduction. We have revised the introduction to follow a more logical flow as you recommended. It now begins with a discussion of the general need for pedagogical skills among health professionals, then addresses the existing strategies in different European countries, and finally focuses on the specific gap related to rehabilitation professionals in Italy. We believe this reorganization enhances the clarity and coherence of the introduction, aligning well with the study’s objectives. Thank you again for your insightful feedback.

It would be beneficial to highlight how your study's findings could contribute to the improvement of educational programs for health professionals, not just in Italy but potentially in other similar contexts.

**Authors’ reply:** Thank you for this suggestion. Following your feedback we have added a paragraph to conclusion section highlighting how our findings could contribute to the improvement of Italian and not educational programmes for healthcare professionals.

**Authors’ action:**

**Page 14, lines 439-442:** Our findings highlight the potential benefits of pedagogical programmes focused on active learning, interdisciplinary collaboration, and communication skills for healthcare professionals in rehabilitation. These findings lay a foundation for future research on investigating the experience of pedagogical programmes in other countries to form updated curricula for rehabilitation healthcare professionals.

When referring to 'Northern ones', consider using 'Northern European countries' or a more precise term to maintain clarity and consistency.

**Authors’ reply:** Amended as suggested

Some of the cited literature in the introduction section seems foundational but might be a bit dated. Including more recent studies or reviews on pedagogical training for health professionals, if available, could enhance the introduction's relevance.

**Authors’ reply:** Thank you for your suggestion to include more recent studies in the introduction. We have reviewed and updated the literature, incorporating recent studies and reviews on pedagogical training for health professionals where available. We believe these additions enhance the introduction's relevance and strengthen the context for our study

How was the decision made regarding the number of focus groups (three groups) and the size of each (5-6 participants)? Was this based on a saturation point or other methodological considerations?

**Authors’ reply:** We would like to thank the reviewer for seeking clarification on the sample size. We have reported that we adopted the ‘information power’ and not ‘data saturation’ as it is inappropriate for RTA. Please, see below the new paragraph.

**Authors’ action:**

**Pages 4,5 lines 122-130:** The focus groups were structured to increase variability inside them. The sample size was guided by the concept of ‘information power’ [11] rather than the commonly used but methodologically inappropriate for RTA ‘data saturation’ [12]. According to the information power model, the number of participants needed depends on factors such as the study aim, sample specificity, theoretical background, quality of dialogue, and analysis strategy [11]. Given the expertise of the researchers in qualitative research and pedagogy, the solid theoretical foundations of our study, the specificity of our research question, and the purposeful selection process, an estimate of 16 to 18 participants divided into three focus groups was considered, including at least one individual with clinical expertise, one with lecturing experience, and one with management/coordination experience per each FGD. Furthermore, the authors aimed to diversify the group by considering participants' gender, age, and years of enrolment in the MSc.

The study uses a 'dual moderator' focus group approach. Could you clarify why this particular method was chosen?

**Authors’ reply:** Thank you for highlighting this. We chose to have two moderators to facilitate the progression of the focus group interview, ensuring that all topics were covered and thoroughly investigated. For instance, one moderator (IC), with expertise in qualitative research, ensured that the focus group adhered to the correct procedures, while the other (BM) followed the interview-guide to ensure all content was addressed. It has been specified in the methods section.

**Authors’ action:**

**Page 5, lines 140-144:** The ‘dual moderator’ focus group included eight open-ended questions to investigate the participants' experience of pedagogical courses [21]. One moderator (IC), with expertise in qualitative research, ensured that the focus group adhered to the correct procedures, while the other (BM) followed an interview guide to ensure all contents were addressed. Together, the moderators facilitated the progression of the focus group interview, ensuring that all topics were thoroughly investigated [22].

How were the online focus groups managed to ensure engagement and minimize potential distractions or technical issues? Were any strategies implemented to maintain the quality of interaction?

**Authors’ reply:** Thank you for your insightful questions regarding the management of the online focus groups. We appreciate your concerns about ensuring engagement and minimizing potential distractions or technical issues.

In our study, we did not implement specific strategies or have dedicated support to address these challenges. Participants were informed about the online platform beforehand, and while we encouraged open communication and participation, we relied on the inherent motivation of the participants to engage in the discussions.

We acknowledge that this approach may not have maximized engagement or minimized distractions, and it is an area we recognize could be improved in future research. We appreciate your feedback, which will help us enhance the quality of online focus group management in future studies.

Why was Braun and Clark's Reflexive Thematic Analysis (RTA) selected for this study?

**Authors’ reply:** Thank you for your question regarding our choice of Braun and Clarke's Reflexive Thematic Analysis (RTA) for this study. We selected RTA for several key reasons:

1. **Flexibility and Adaptability:** RTA is a highly flexible approach that can be adapted to various research questions and types of qualitative data. This flexibility was crucial for our study, as we sought to explore the nuanced experiences of participants in pedagogical courses.
2. **Focus on Participants' Experiences:** RTA allows for a deep exploration of participants' experiences and the meanings they attach to those experiences. This aligns with our study's goal of understanding the perceived knowledge and skills gained by health professionals attending pedagogical courses.
3. **Inductive Approach:** RTA emphasizes an inductive approach, which involves generating themes directly from the data rather than imposing pre-existing theories or frameworks. This aspect was essential for capturing the authentic voices of the participants and ensuring that the themes accurately reflected their perspectives.
4. **Rich Data Interpretation:** RTA enables a detailed and rich interpretation of qualitative data, allowing us to construct a comprehensive narrative of participants' experiences. This depth of analysis is vital in the context of pedagogical training, where understanding the subtleties of participants' insights can inform future educational strategies.
5. **Collaborative Analysis:** The RTA framework facilitated collaborative analysis among the research team, promoting diverse perspectives during the coding and theme development process. This collaborative approach helped enhance the rigor and validity of our findings.

Overall, Braun and Clarke's Reflexive Thematic Analysis provided the appropriate framework for our study, enabling us to generate meaningful themes that reflect the participants' lived experiences in a rigorous and nuanced manner.

Thank you for your interest in our methodology

**Authors’ action:**

**Page 7, lines 180-190:** Qualitative data were analysed using Braun and Clark's ‘Reflexive Thematic Analysis’ (RTA) to identify the patterns of meaning and consequently generate themes relating to the experiences of the pedagogical courses [23]. RTA was selected for analysing the qualitative data due to its flexibility and adaptability, which allowed us to capture the nuanced perspectives of participants in pedagogical training courses. RTA is particularly suitable for studies with an experiential focus, as it enables a deep exploration of participants' personal insights and the meanings they attach to these experiences. This approach allowed us to generate themes inductively, ensuring that they authentically reflected participants' voices without imposing pre-existing frameworks. Moreover, RTA facilitated a collaborative analysis process, enriching the rigor of theme development and aligning with the social constructionist framework of this study. From the point of view of the epistemological framework, this study adopted a social constructionist approach and an experiential orientation. The coding was mainly at a descriptive/semantic level. To analyse the focus group data, we followed the six steps of RTA as outlined by Braun and Clarke, as shown in Table 2.

How did you determine the naming of each theme (e.g., 'A Brave New Pedagogical World')? Were these names derived directly from participants' language, or did they emerge from the researchers' interpretation of the data?

**Authors’ reply:** Thank you for your insightful question regarding the naming of the themes. The names of each theme, such as "A Brave New Pedagogical World," were developed collaboratively by the research team during the data analysis process.

As outlined in the *Data Analysis* section, we followed Braun and Clarke's Reflexive Thematic Analysis (RTA), which included several phases. After familiarizing ourselves with the data and generating initial codes, we clustered similar codes to identify patterns and relationships. The naming of the themes emerged during the phase of defining and refining them, where we aimed to ensure that each name accurately represented the participants' experiences and perspectives.

While some theme names were influenced by the language used by participants, they were also shaped by our interpretation of the data to effectively convey the essence of the discussions. We engaged in peer debriefing and reached a consensus as a team to finalize the theme names, ensuring they reflected the core narratives shared by participants during the focus groups. We appreciate your interest in this aspect of our analysis and hope this explanation clarifies our process.

You mention the transformative experience that participants had regarding pedagogy. Consider expanding on how this experience aligns with adult learning theories (e.g., transformative learning theory or experiential learning theory). This can strengthen the theoretical underpinning of your findings.

**Authors’ reply:** Thank you for this suggestion, which we agree have strengthened the discussions. We have implemented a new paragraph in the discussion section to discuss how our findings resonate with the adult learning theories.

**Authors’ action:**

**Page 13, lines 387-394:** Moreover, the transformative experience reported by participants aligns with adult learning theories such as the “Transformative Learning Theory” and the “Experiential Learning Theory” [33,34]. “Transformative Learning Theory” emphasises ‘critical reflection’ to reconsider pre-existing beliefs and attitudes about one’s profession aiming at taking informed action and implementing new knowledge [33]. “Experiential Learning Theory”, rooted in constructivist philosophy, highlights the role of interpersonal interactions and contextual learning as fundamental to experiential learning [34]. This approach enables learners to translate, or refine, learning in new contexts to meet new challenges [34]. For instance, participants reported shifting what they learnt on pedagogy into their clinical practice.
